# Cardiac, mandibular and thymic phenotypical association indicates that cranial neural crest underlies bicuspid aortic valve formation in hamsters

**DOI:** 10.1371/journal.pone.0183556

**Published:** 2017-09-27

**Authors:** Jessica Martínez-Vargas, Jacint Ventura, Ángela Machuca, Francesc Muñoz-Muñoz, María Carmen Fernández, María Teresa Soto-Navarrete, Ana Carmen Durán, Borja Fernández

**Affiliations:** 1 Departament de Biologia Animal, Biologia Vegetal i Ecologia, Facultat de Biociències, Universitat Autònoma de Barcelona, Cerdanyola del Vallès, Spain; 2 Departamento de Biología Animal, Facultad de Ciencias, Universidad de Málaga, Málaga, Spain; 3 Instituto de Investigación Biomédica de Málaga (IBIMA), Málaga, Spain; 4 CIBERCV Enfermedades Cardiovasculares, Málaga, Spain; Harvard Medical School, UNITED STATES

## Abstract

Bicuspid aortic valve (BAV) is the most prevalent human congenital cardiac malformation. It may appear isolated, associated with other cardiovascular malformations, or forming part of syndromes. Cranial neural crest (NC) defects are supposed to be the cause of the spectrum of disorders associated with syndromic BAV. Experimental studies with an inbred hamster model of isolated BAV showed that alterations in the migration or differentiation of the cardiac NC cells in the embryonic cardiac outflow tract are most probably responsible for the development of this congenital valvular defect. We hypothesize that isolated BAV is not the result of local, but of early alterations in the behavior of the NC cells, thus also affecting other cranial NC-derived structures. Therefore, we tested whether morphological variation of the aortic valve is linked to phenotypic variation of the mandible and the thymus in the hamster model of isolated BAV, compared to a control strain. Our results show significant differences in the size and shape of the mandible as well as in the cellular composition of the thymus between the two strains, and in mandible shape regarding the morphology of the aortic valve. Given that both the mandible and the thymus are cranial NC derivatives, and that the cardiac NC belongs to the cephalic domain, we propose that the causal defect leading to isolated BAV during embryonic development is not restricted to local alterations of the cardiac NC cells in the cardiac outflow tract, but it is of pleiotropic or polytopic nature. Our results suggest that isolated BAV may be the *forme fruste* of a polytopic syndrome involving the cranial NC in the hamster model and in a proportion of affected patients.

## Introduction

The aortic valve of mammals is the anatomical structure responsible for preventing blood reflux from the aorta to the left ventricle. It normally holds three leaflets or cusps and thus is named tricuspid aortic valve (TAV). In humans, congenital malformations of the aortic valve may cause clinically relevant valve malfunction. The most frequent congenital aortic valve malformation is the bicuspid aortic valve (BAV), which is indeed the most prevalent human cardiac malformation, with an incidence of 0.5–2% in the general population [1–3]. Although a BAV may be clinically silent during the lifetime of the carrier, it entails a high risk of valvulopathies and aortopathies [4]. A BAV may appear isolated, associated with other cardiovascular malformations, e.g. ventricular septal defect and coarctation of the aorta [5,6], or forming part of syndromes, e.g. Turner, DiGeorge, hypoplastic left heart, Bosley-Salih-Alorainy, Athabascan Brainstem Dysgenesis, Loeys-Dietz, Andersen-Tawl, Larsen, Alagille and Kabuld [7–9]. Altogether, it has been estimated that BAV disease is responsible for more human deaths than the sum of all the other known congenital cardiac malformations [10].

Two types of BAV have been described depending on the anatomical position of the leaflets, i.e. the antero-posterior (A-P) and the latero-lateral (L-L) types. Each type can present two forms (subtypes) depending on the absence (subtype 0) or presence (subtype 1) of a raphe [11]. Although it has been shown that each anatomical type of BAV has a distinct etiology [12], and it is assumed that multiple etiological mechanisms of BAV development may exist [8,9], experimental studies in rodent models have revealed that both types of isolated BAV can be formed by alterations in the behavior of the cardiac neural crest cells [12,13].

The neural crest (NC) is a transient, migratory, and pluripotent embryonic cell population that develops from the dorsal neural tube. Once at their final destinations, NC cells (NCCs) differentiate into a wide range of cell types and tissues [14–18]. The NCCs that specifically originate from midbrain to the third somite levels of the dorsal neural tube are known as cranial NCCs (CrNCCs) [14]. These cells form most of the mesenchymal structures of the skull, maxilla, mandible, neck, and pharyngeal organs such as the thymus. A set of CrNCCs known as cardiac NCCs (CaNCCs) contribute to the development of the cardiac outflow tract and the arteries of the aortic arch system [19–22].

Craniofacial and cardiac malformations are among the most common birth defects in humans, the pathogenesis of which often involves CrNCCs alterations causing polytopic syndromes [21]. The anomalies of the cardiac outflow tract and the aortic arch system associated with syndromic BAV are considered manifestations of a spectrum of disorders involving the head and neck region caused by NCCs defects [23]. Indeed, the extracardiac defects associated with syndromic BAV affect anatomical structures derived from the CrNCCs, like the thymus, the parathyroid glands, the mandible, and the palate, among others [7–9,24–28]. In addition, genetic manipulation in mice has shown that absence of *Hoxa1* expression in NCCs causes syndromic BAV associated with defects of the cardiac outflow tract, aortic arch, thymus, parathyroid glands, and craniofacial structures [29]. Regarding isolated BAV development, studies with a spontaneous animal model have revealed that BAV formation relies on the abnormal behavior of NCCs colonizing the embryonic cardiac outflow tract, thus assuming that BAV results from local alterations of CaNCCs migration [12].

Given that 1) the pathogenetic substratum of many cases of isolated BAV formation is an altered behavior of the CaNCCs migrating into the cardiac outflow tract, 2) knocking out NC specific genes results in syndromic BAV, and 3) cardiac and extracardiac defects associated with syndromic BAV result from CrNCCs alterations, we hypothesize that the causal defect leading to isolated BAVs during embryonic development might not be restricted to local alterations of the CaNCCs, but could be of pleiotropic or polytopic nature, affecting different CrNC-derived structures. It could then be expected that BAV carriers present undetected alterations of CrNC-derived structures.

The mandible of the current mammalian species is formed by a single bone, the dentary, which originates from cells that migrate from the posterior mesencephalic NC (see [30], and references therein). Once in the first mandibular arch, these NCCs differentiate into six major morphogenetic units (the ramus, the molar and incisor alveolar components, and the coronoid, condylar and angular processes) and Meckel’s cartilage [31–33]. Two primary functional modules, derived from these morphogenetic units, have been recognized (see [34] and references therein, [35,36]): a distal module bearing the teeth (alveolar region), and a proximal module that articulates with the skull case and constitutes the attachment area for most of the masticatory muscles (ascending ramus). The study of the genetic basis of mouse mandible shape has revealed that genetic modularity also occurs in this structure, in the same way as functional modularity does [37–39].

The thymus is a lymphoepithelial organ composed of connective tissue, lymphocytes, macrophages, reticulo-epithelial cells, and dendritic cells [40,41]. The thymic primordium develops as a result of interactions between the third pharyngeal pouch endoderm and surrounding NC mesenchyme originating at the hindbrain level. This primordium is subsequently encapsulated by NCCs, which promote early growth, patterning, differentiation, and proliferation [42]. Once lymphopoiesis is established in the thymus, some NC-derived reticulo-epithelial cells undergo hypertrophy and get organized in concentric layers of squamous-looking epithelial cells called Hassall’s corpuscles [43–46]. Although Hassall’s corpuscles were initially suggested to be the “cemetery” of degenerated thymocytes, currently it is known that they also participate in thymocyte maturation, as well as in secretion of cytokines and growth factors [41,47,48].

In this study, we aim to assess whether morphological variation of the aortic valve is linked to phenotypic variation of the mandible and the thymus, both derived from the CrNCCs. To this end, we use a well-established Syrian hamster (*Mesocricetus auratus*) model of isolated A-P BAV, and control hamsters. BAVs in the hamster model correspond to the human A-P type (Sievers subtypes 0 and 1) [11]. To compare the phenotypic variation of the mandible between these groups of animals, we apply geometric morphometrics (see [49] and references therein), a powerful analytical method for determining changes in shape independent of the effects of size. To compare thymic phenotypic variation, we examine the general anatomy and histology of the thymus in both groups of animals, and quantify the Hassall’s corpuscles, a direct NC derivative in the thymus.

## Materials and methods

### Animals

The animals used in the present study belonged to two unrelated Syrian hamster lines: one inbred (T; n = 101) and one outbred (H; n = 57) strain. The T strain shows a high incidence (∼40%) of A-P BAV, resulting from a systematic selective inbreeding by mating affected siblings. The characteristics of this unique strain have been published elsewhere [12,50,51]. The H strain was used to obtain control animals. It derives from a closed colony of hamsters that has been outbred since 1990 and commercialized by Janvier (France) (code RjHam: AURA).

The animals were handled in accordance with the European and Spanish guidelines for animal welfare, and with the recommendations in the Guide for the Care and Use of Laboratory Animals of the National Institute of Health. The protocol was approved by the Ethics Committee of Animal Experiments of the University of Málaga (CEUMA; Ethics authorization number: 2015–0006). The animals were housed in standard cages, fed chow and supplied with water *ad libitum*, and sacrificed by CO_2_ inhalation. After death, the head was removed, the chest was opened, and the thymus and heart were dissected out. Since no sexual dimorphism in the size and shape of the mandible, thymus, and aortic valve was detected in preliminary analyses, data of males and females were analyzed together.

### Aortic valve

The aortic valve of each specimen was exposed by dissection under the binocular microscope, and its morphology assessed. In order to document the valve morphologies, the aortic valves of some specimens were subsequently analyzed by scanning electron microscopy as previously described [52]. Briefly, each specimen was fixed by immersion in 1% paraformaldehyde and 2% glutaraldehyde in 0.005 M sodium cacodylate buffer (pH 7.3) for several hours, rinsed with the same buffer, dehydrated with increasing concentrations of ethanol, dried by the critical point method, and gold sputter coated. Observations were made using a *Jeol JSM-840* scanning electron microscope.

**Fig 1:**
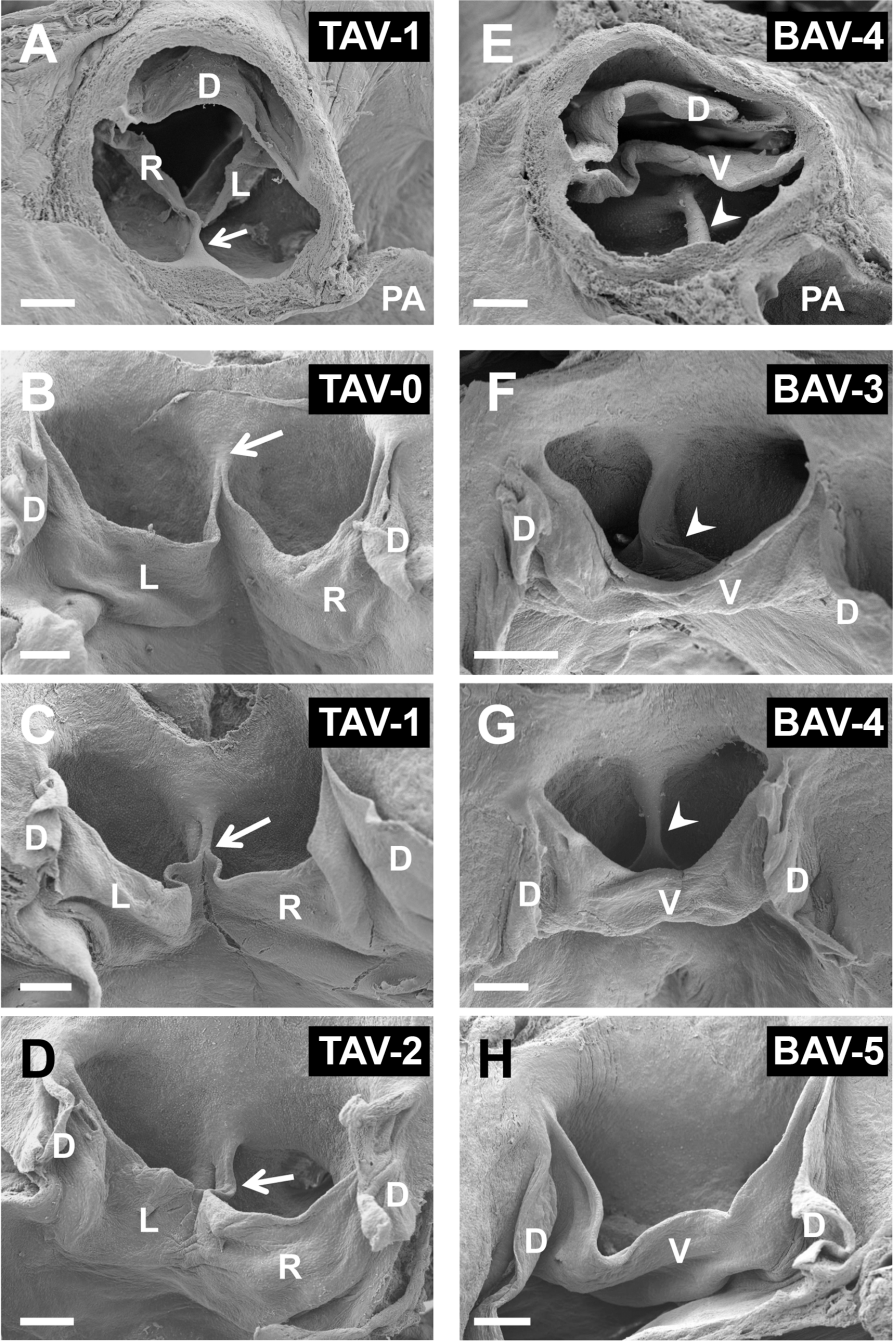
**Scanning electron micrographs of tricuspid (A-D) and bicuspid (E-H) aortic valves of hamsters, with the different morphological variants.** (A,C) TAV-1; (B) TAV-0; (D) TAV-2; (E,G) BAV-4; (F) BAV-3; (H) BAV-5. Panels A and E show cranial views, whereas B-D and F-H show frontal views after dissecting the dorsal leaflet. The arrows point to the ventral commissure, and the arrowheads to the raphes. D, L, R, and V: dorsal, left, right, and ventral leaflets, respectively. PA: pulmonary artery. Scale bars: 200 μm.

Previous studies with the hamster model of A-P BAV showed that, similar to humans, several intermediate morphotypes between pure tricuspid and bicuspid aortic valve morphologies exist. In the hamster model, this morphological variability relies on the degree of fusion of the right and left leaflets and the presence and size of the raphe [51–54]. It has been shown that all these morphotypes result from the degree of severity of the underlying morphogenetic defect [12,50–54]. Accordingly, for the study of the association between mandibular and aortic valve phenotypes, six categories were established for the aortic valve morphology [50–54] (Fig 1). The specimens classified as having TAV were further grouped according to the degree of fusion of the valve leaflets: TAV without fusion (TAV-0); TAV with little fusion (TAV-1); TAV with large fusion (TAV-2). The specimens classified as having BAV were further grouped according to the size of the raphe: BAV with a big raphe (BAV-3); BAV with a small raphe (BAV-4); BAV without a raphe (BAV-5). The morphological variant of the aortic valve could not be assessed in one individual.

### Mandible

For the study of the association between mandibular and aortic valve phenotypes, a total of 132 adult specimens were used and grouped according to: a) the strain: H strain (n = 42), T strain (n = 90); b) the strain and the aortic valve phenotype: H strain with TAV (n = 42), T strain with TAV (n = 59), T strain with BAV (n = 31); and c) the strain and the morphological variant of the aortic valve: H strain with TAV-0 (n = 13), H strain with TAV-1 (n = 24), H strain with TAV-2 (n = 4), T strain with TAV-0 (n = 4), T strain with TAV-1 (n = 22), T strain with TAV-2 (n = 33), T strain with BAV-3 (n = 8), T strain with BAV-4 (n = 5), T strain with BAV-5 (n = 18). The morphological variant of the aortic valve could not be assessed in one individual of H strain.

**Fig 2:**
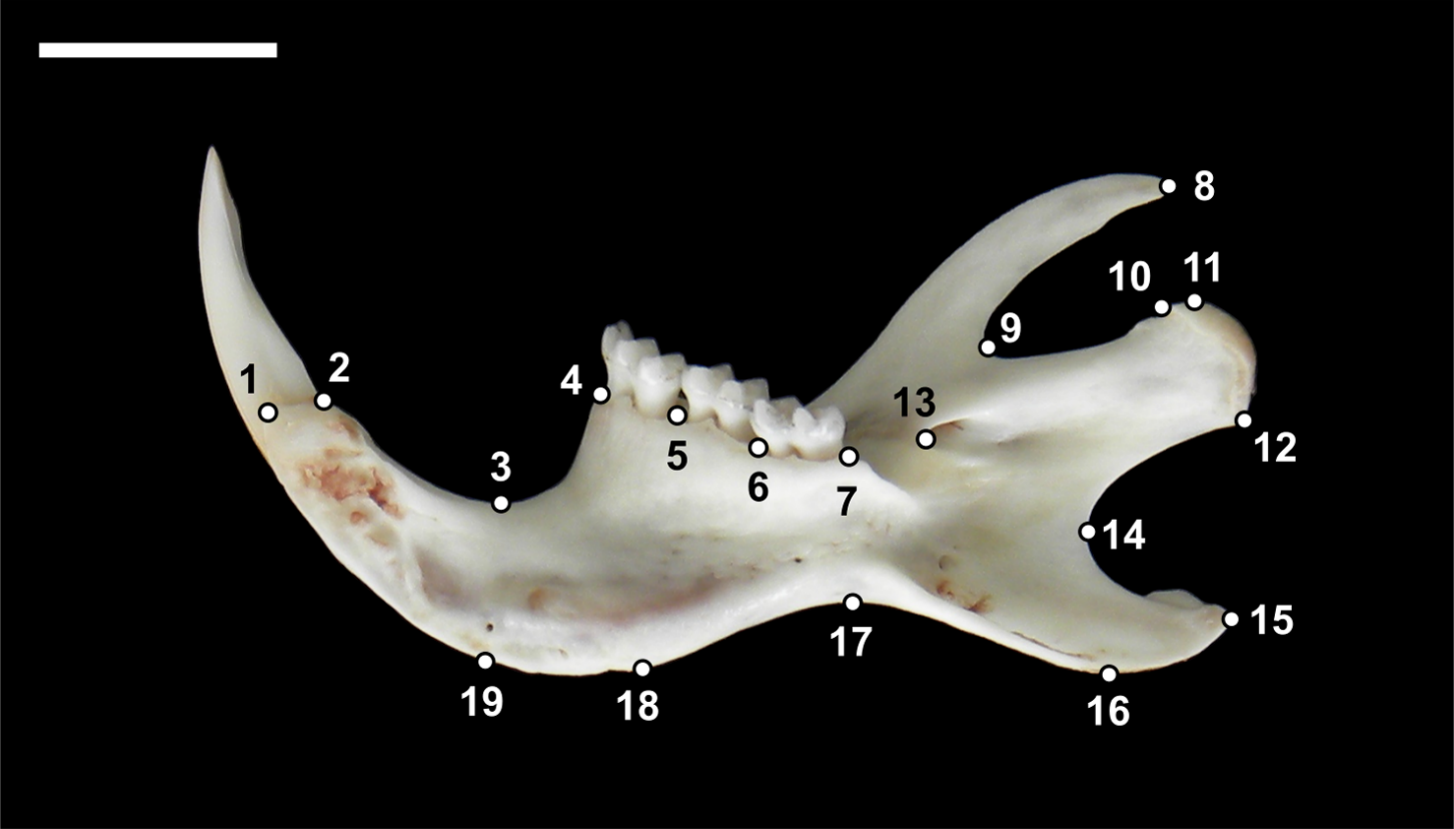
Lingual view of a right hemimandible with the layout of the 19 landmarks used in the geometric morphometric analyses.

Mandibles of all individuals were dissected and cleaned by hand, and their two halves were separated at the mandibular symphysis. Images of the lingual side of the 264 right and left hemimandibles, together with a scale bar, were obtained with a *Nikon COOLPIX P90* digital camera. Nineteen two-dimensional landmarks were digitized twice in each scaled image by the same observer (Fig 2, S1 Table), using the tpsDig2 software [55]. The morphometric study was approached using the geometric morphometric methods implemented in the MorphoJ package, ver. 1.06d [56].

Size of all hemimandibles was estimated through the centroid size (CS) [57]. Landmark configurations of the left hemimandibles were mirrored. Landmark coordinates of all hemimandibles were superimposed through a generalized Procrustes fit and projected onto the shape tangent space [57,58]. This procedure allowed the extraction of the shape information of each landmark configuration as Procrustes coordinates, which consist in the landmark coordinates devoid of any variation due to size, position, and orientation [57].

The data were split into two datasets according to strain (H and T) in order to examine their patterns of mandible form variation separately. The dataset corresponding to strain T was further subdivided according to aortic valve phenotype (BAV and TAV) in order to assess form variation in each case. To omit the influence of the aortic valve phenotype when comparing the two strains, an additional dataset was created by grouping the individuals with TAV from each strain. Multivariate statistical analyses were conducted on the entire sample and each dataset.

CS and Procrustes coordinates were respectively subjected to two-factor and Procrustes analyses of variance (ANOVAs) [58,59]. This procedure allowed the partitioning of total variation into the components of symmetric and asymmetric variation. The symmetric component accounts for the variation among individuals in the averages of landmark configurations of the right and left sides. The asymmetric component accounts for the variation within individuals in the differences between landmark configurations of the right and left sides [59,60]. Individual and side were respectively chosen as the random and fixed main effects in the ANOVAs. The individual factor accounted for variation among individuals, and so represented the symmetric component of variation. The side factor accounted for directional asymmetry (DA), i.e. the average difference between the two sides. The interaction between both factors estimated fluctuating asymmetry (FA), i.e. the variability of left-right differences among individuals. This interaction term represented the asymmetric component of variation [56,58,61]. Strain, aortic valve phenotype, and morphological variant of the aortic valve were selected as the additional main effects in the ANOVAs conducted on the whole sample. The effect of the morphological variant of the aortic valve was tested in all ANOVAs, while the effect of the aortic valve phenotype was assessed in the ANOVAs conducted on T strain. Strain was chosen as an additional main effect in the ANOVAs performed on the dataset grouping all individuals with TAV. Measurement error was quantified as the residual variation between replicates [58]. Subsequent analyses were conducted only for the symmetric component of variation.

Allometry, the dependence of shape on size, was evaluated with multivariate regressions of shape onto CS. Regressions conducted with the whole sample and the set of individuals with TAV were pooled within strains. Statistical significance was tested using permutation tests with 10,000 iterations under the null hypothesis of no allometric relationship [62,63]. Since a significant dependence of shape on size was always found (see *Mandible*. *Allometry* section), subsequent analyses were based on the covariance matrices obtained from raw data, but also on those obtained from the regression residuals in order to correct for allometry [34]. However, only the results derived from size-corrected data are displayed.

Patterns of shape variation were explored with the principal component analysis (PCA) [64]. The eigenvalues or percentages of total shape variation explained by each principal component (PC), and the graphical distribution of the individual PC scores, were obtained.

Canonical variate analyses (CVAs) and discriminant function analyses (DFAs) were conducted to assess morphological distances among strains, aortic valve phenotypes, and morphological variants of the aortic valve, as well as to examine the shape features that best distinguished between groups in each case. The graphical distribution of the individual CV scores was obtained for combinations of variables (e.g., strain and aortic valve phenotype). Within T strain, a CVA was performed to evaluate the distinct morphological variants of the two aortic valve phenotypes jointly. Furthermore, two different CVAs were conducted to evaluate the morphological variants of BAV and TAV of T strain separately, in order to avoid interaction. Mahalanobis distances (MDs) between pairs of groups were obtained, together with the statistical significance resulting from permutation tests with 10,000 iterations.

### Thymus

For the study of the association between thymic and aortic valve phenotypes, 26 young adult animals (90 to 150 days) from the H (n = 15) and the T (n = 11) strains were used. Young animals were selected in order to avoid possible errors derived from tissue changes associated with thymic involution.

The thymus was dissected together with the aortic arch, cleaned from fat and vessels under the binocular microscope, and photographed, together with a scale bar, using a *Leica DFC 500* camera. The total length and width of each thymic lobe was measured. The samples were fixed by immersion in 4% paraformaldehyde overnight, dehydrated, and embedded in Histosec (Merck KGaA; Darmstadt, Germany). Serial sections longitudinally cut at 7 μm were stained with Delafield’s haematoxylin-eosin (HE) or Masson trichrome, or immunostained using polyclonal antibodies raised against the Hassall’s corpuscle specific cytokeratin-10 (KRT10; AV41730, Sigma). The immunohistochemical method has been described elsewhere [65]. Anti-KRT10 (1:200; Sigma) and biotin-conjugated anti-rabbit IgGs (1:250) were used as primary and secondary antibodies, respectively. The sections were observed with a *Leica DMSL* light microscope. Images were acquired using a *Leica DFC 500* camera.

In order to test for possible differences in symmetry between the thymic lobes of each animal, and in the dimensions of the thymus between strains, the volume of each thymic lobe was estimated and compared. The volume (V) of each thymic lobe was estimated using the mathematical formula of the ovoid volume: *V* = *R*1 × *R*2 × Π where R1 and R2 (major and minor radii respectively) correspond to half the length and half the width of the thymus respectively. First, the volumes of the right and left lobes of each animal were compared. Next, the mean volume of the right and left lobes of each animal was calculated and normalized to the total length of each animal. Differences in volume between the right and left thymic lobes and between H and T strains were assessed by means of Student’s t-test.

The Hassall’s corpuscles were quantified in histological sections stained with HE, corresponding to the central portion of each thymic lobe. The number of corpuscles was counted in optic fields at a magnification of 200X. A minimum of 20 optical fields in at least six different sections per specimen were analyzed. Then, the mean number of Hassall’s corpuscles per square millimeter was calculated for each lobe. Differences in density of Hassall’s corpuscles (number of corpuscles per mm^2^) between the right and left thymic lobes of each animal, and between H and T strains, were assessed by means of Student’s t-test.

## Results

### Mandible. Sources of size and shape variation

The ANOVAs carried out on the whole sample revealed significant individual variation and fluctuating asymmetry in mandible shape and size, but significant directional asymmetry only in mandible shape (Tables 1 and 2). Variation resulting from measurement error was negligible, since it was significantly exceeded by variation in fluctuating asymmetry (Tables 1 and 2). The two strains of hamsters significantly differed in mandible size and shape (Tables 1 and 2). Mean CS was significantly higher in the H strain than in the T strain.

**Table 1:** Two-factor ANOVA for centroid size conducted on the entire sample.

	CENTROID SIZE				
Effect	SS	df	MS	F	*P*
Individual	9.373	123	7.621 x 10^−2^	58.88	<0.001
Side	0.003	1	2.811 x 10^−3^	2.17	0.143
Individual × Side	0.170	131	1.294 x 10^−3^	25.57	<0.001
Strain	8.628	1	8.628	113.22	<0.001
AV phenotype	0.197	1	0.197	2.58	0.111
AV morphological variant	0.483	6	0.080	1.06	0.393
Measurement error	0.013	264	5.1 x 10^−5^		

SS, sum of squares; df, degrees of freedom; MS, mean squares; F, F statistic; *P*, *P*-value; AV, aortic valve.

**Table 2:** Procrustes ANOVA for shape conducted on the entire sample.

	SHAPE						
Effect	SS	df	MS	F	*P*	Pillai’s tr	*P*
Individual	0.405	4182	9.692 x 10^−5^	2.72	<0.001	21.97	<0.001
Side	0.008	34	2.303 x 10^−4^	6.46	<0.001	0.83	<0.001
Individual × Side	0.159	4454	3.564 x 10^−5^	14.97	<0.001	28.28	<0.001
Strain	0.151	34	4.453 x 10^−3^	45.95	<0.001	0.97	<0.001
AV phenotype	0.002	34	5.533 x 10^−5^	0.57	0.979	0.30	0.312
AV morphological variant	0.015	204	7.254 x 10^−5^	0.75	0.997	1.22	0.998
Measurement error	0.021	8976	2.381 x 10^−6^				

SS, sum of squares; df, degrees of freedom; MS, mean squares; F, F statistic; *P*, *P*-value; Pillai’s tr, Pillai’s trace; AV, aortic valve.

The ANOVAs conducted on the dataset grouping all specimens with TAV also displayed significant differences in mandible size and shape between strains H and T (F = 82.78, *p*<0.001 and Pillai’s trace = 0.98, *p*<0.001, respectively). A significant effect of aortic valve phenotype on mandible shape was detected in the T strain (Pillai’s trace = 0.54, *p*<0.05). No significant effect of the morphological variant of the aortic valve was detected on mandible size or shape in any dataset.

### Mandible. Allometry

Symmetric shape variation significantly depended on size in both strains of hamsters (*p*<0.001), although H strain showed a slightly higher proportion of shape variation accounted for by size variation (H: 12.50%; T: 10.95%). Within T strain, individuals both with TAV and BAV showed a significant allometric relationship (*p*<0.001), but a slightly greater percentage of allometry corresponded to specimens with TAV (TAV: 14.99%; BAV: 11.52%).

### Mandible. Patterns of shape variation and differentiation

**Fig 3:**
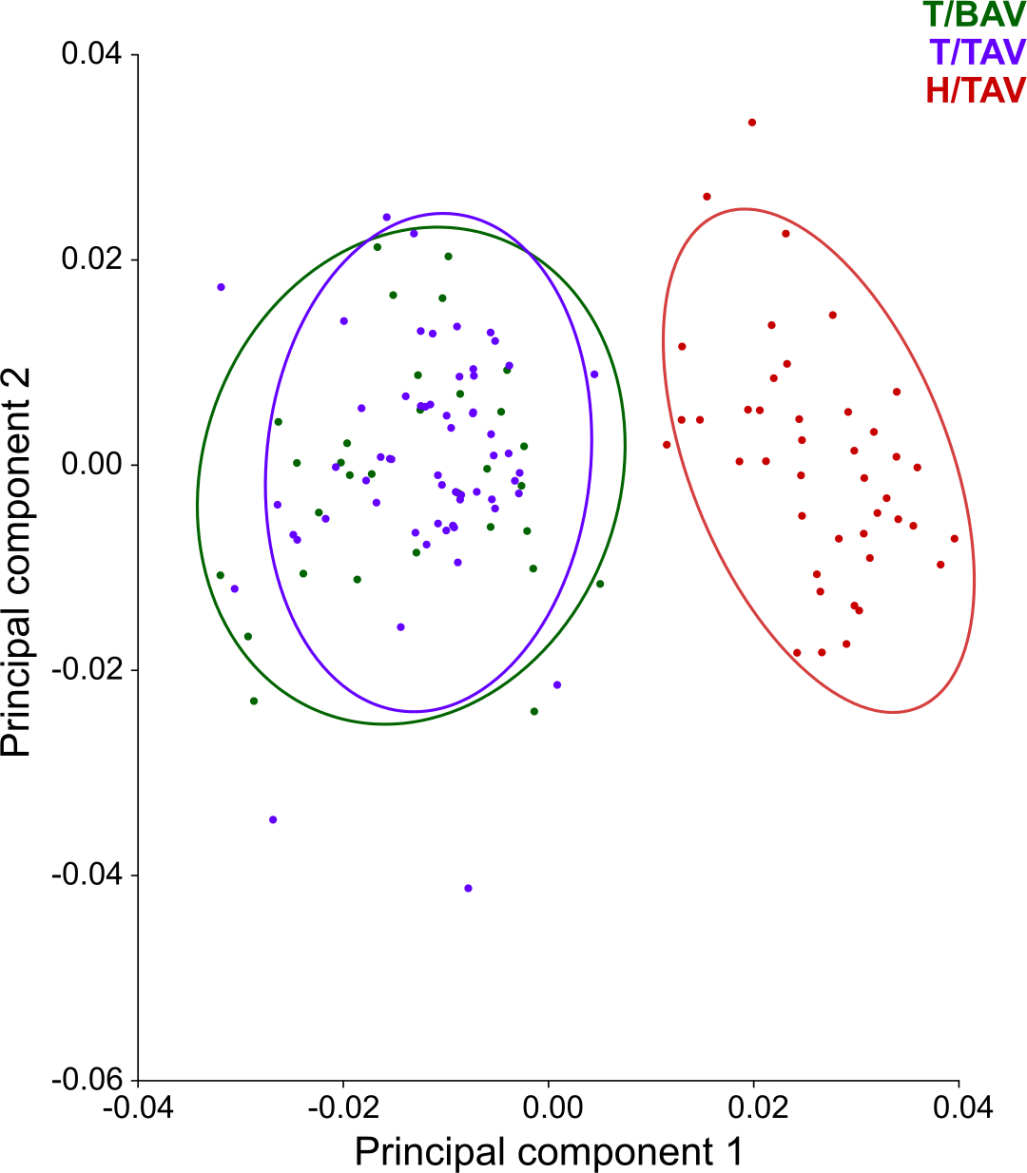
Scatter plot of PC1 *vs*. PC2 scores according to strain and aortic valve phenotype. The PC1 axis explains 36.63% of total shape variation, and differentiates between the two strains.

The two strains of hamsters showed a non-overlapped arrangement in the scatter plot of PC1 *vs*. PC2 scores resulting from the PCA conducted with the entire sample (Fig 3). The PC1 *vs*. PC2 scores of the specimens with BAV and TAV belonging to T strain showed a mixed arrangement (Fig 3). According to the eigenvalues, PC1 and PC2 accounted for 36.63% and 11.99% of total shape variance, respectively.

**Fig 4:**
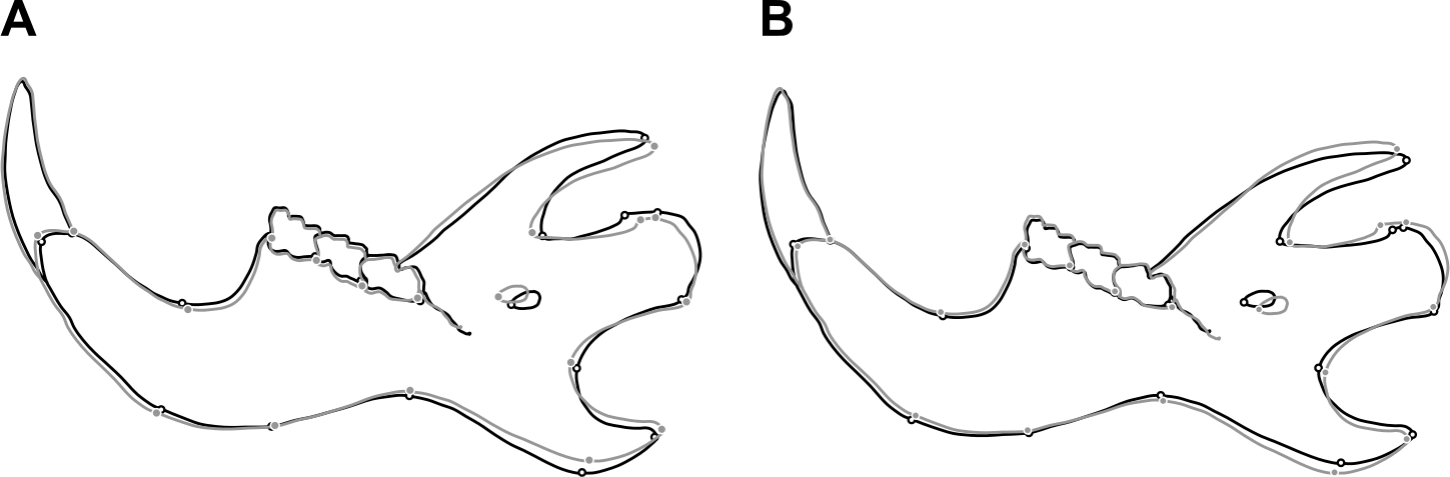
Diagrams of mean mandible shape differences. (A) Mean mandible shape difference between H and T strains. Black: Mean shape of H strain. Grey: Mean shape of T strain. Scale factor: 1.0. (B) Mean mandible shape difference between BAV and TAV phenotypes. Black: Mean shape of BAV phenotype. Grey: Mean shape of TAV phenotype. Scale factor: 2.0.

The CVA and DFA also revealed the existence of shape differences between the two strains of hamsters. The MD between the two strains was statistically significant (MD = 12.916, *p*<0.001). Mandible shape differences between the means of H and T strains mainly involved the ascending ramus (Fig 4A).

**Fig 5:**
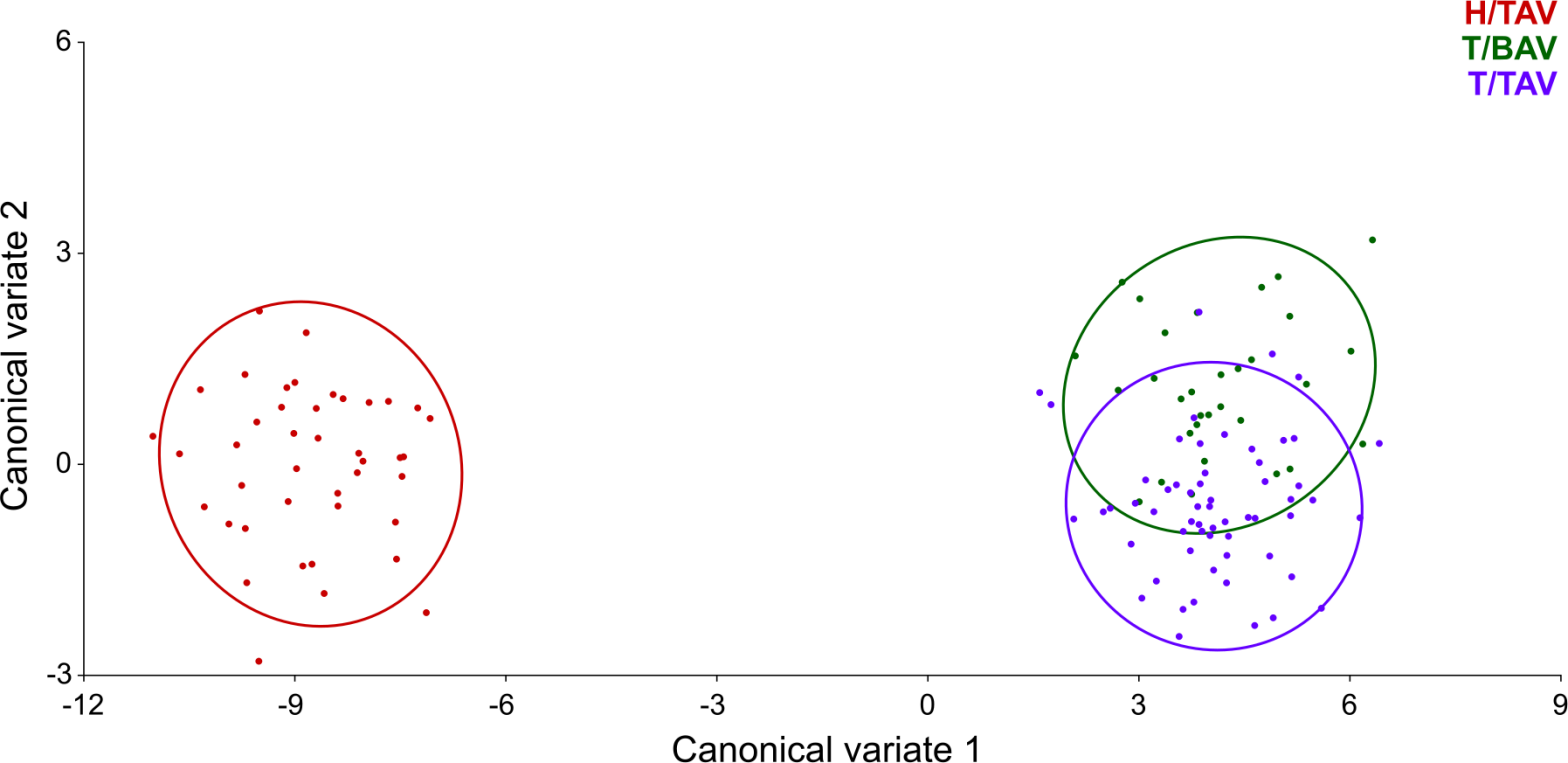
Scatter plot of CV1 *vs*. CV2 scores according to strain and aortic valve phenotype. The CV1 axis especially differentiates between the two strains, whereas the CV2 axis differentiates between the specimens with TAV and BAV belonging to T strain.

In the CVA considering both the strain and the aortic valve phenotype for grouping, the CV1 and CV2 axes respectively accounted for 98.75% and 1.25% of shape variation among groups, scaled for the within-group variation. According to the scatter plot of CV1 *vs*. CV2 scores, the greatest morphological distance occurred between the specimens of H strain, with TAV, and the specimens with BAV and TAV belonging to T strain along the CV1 axis (Fig 5). However, certain differentiation in mandible shape was also detected between the specimens with TAV and the individuals with BAV from T strain along the CV2 axis (Fig 5). According to the DFA, 84.85% of the specimens were allocated in the correct phenotypic group. The MD between the individuals with different aortic valve phenotype was statistically significant, both when taking into account the entire sample (MD = 1.967, *p*<0.001) and only T strain (MD = 1.941, *p*<0.001). Differences in mandible shape between the means of the BAV and TAV phenotypes again particularly involved the ascending ramus (Fig 4B).

**Fig 6:**
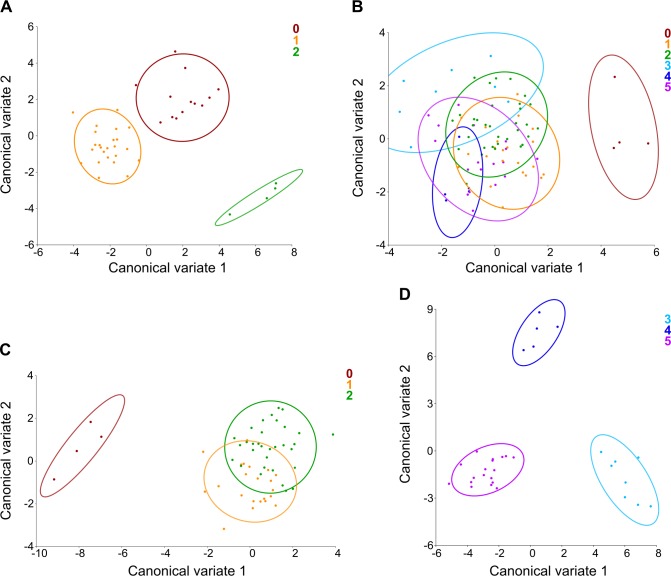
Scatter plots of CV1 *vs*. CV2 scores according to the morphological variants of the aortic valve. (A) Morphological variants of TAV (0–2) in H strain; (B) morphological variants of TAV (0–2) and BAV (3–5) in T strain; (C) morphological variants of TAV (0–2) in T strain; (D) morphological variants of BAV (3–5) in T strain.

In the CVA of H strain, which grouped specimens according to the morphological variants of TAV, 74.65% and 25.35% of shape variation among groups wererespectively explained by CV1 and CV2. The three groups exhibited a non-overlapped distribution in the scatter plot of CV1 *vs*. CV2 scores (Fig 6A). However, a relatively greater isolation of group TAV-2 was detected (Fig 6A). All pairwise MDs were statistically significant (Table 3).

**Table 3:** Mahalanobis distances between the groups with distinct morphological variants of TAV in H strain.

	TAV-0	TAV-1
**TAV-1**	4.921**	–
**TAV-2**	6.954**	8.916**

** *p*<0.001

In the CVA of T strain, where specimens were grouped according to the distinct morphological variants of both TAV and BAV, CV1 and CV2 respectively accounted for 45.09% and 20.59% of shape variation among groups. In the scatter plot of CV1 *vs*. CV2 scores, the six groups generally displayed an overlapped distribution, except for group TAV-0, which was separated along the CV1 axis (Fig 6B). Most of MDs between groups were statistically significant, especially between group TAV-0 and the other groups (Table 4).

**Table 4:** Mahalanobis distances between the groups with distinct morphological variants of BAV and TAV in T strain.

	TAV-0	TAV-1	TAV-2	BAV-3	BAV-4
**TAV-1**	5.176**	–	–	–	–
**TAV-2**	5.360**	1.675	–	–	–
**BAV-3**	6.788*	3.551**	2.986*	–	–
**BAV-4**	6.840*	3.267	3.392*	3.914	–
**BAV-5**	5.805**	2.101	2.096*	3.074*	2.831

* *p*<0.05

** *p*<0.001

When conducting a separate CVA only with the specimens with TAV from T strain, grouping them according to the morphological variants of TAV, 86.47% and 13.53% of shape variation among groups were respectively explained by CV1 and CV2. In the scatter plot of CV1 *vs*. CV2 scores, the groups comprising specimens with some degree of fusion of the aortic leaflets (TAV-1 and TAV-2) appeared partially overlapped, whereas the group of specimens with no fusion of the aortic leaflets (TAV-0) was relatively more isolated along the CV1 axis (Fig 6C). In the CVA of the specimens with BAV from T strain, grouped according to the morphological variants of BAV, CV1 and CV2 respectively accounted for 56.57% and 43.43% of shape variation among groups. In this case, the three groups did not overlap in the scatter plot of CV1 *vs*. CV2 scores (Fig 6D). Pairwise MDs were statistically significant in both cases (Tables 5 and 6).

**Table 5:** Mahalanobis distances between the groups with distinct morphological variants of BAV in T strain.

	BAV-3	BAV-4
**BAV-4**	10.790*	–
**BAV-5**	8.888**	9.406**

* *p*<0.05

** *p*<0.001

**Table 6:** Mahalanobis distances between the groups with distinct morphological variants of TAV in T strain.

	TAV-0	TAV-1
**TAV-1**	8.129**	–
**TAV-2**	8.868**	2.015**

** *p*<0.001

### Thymus. Size

**Fig 7:**
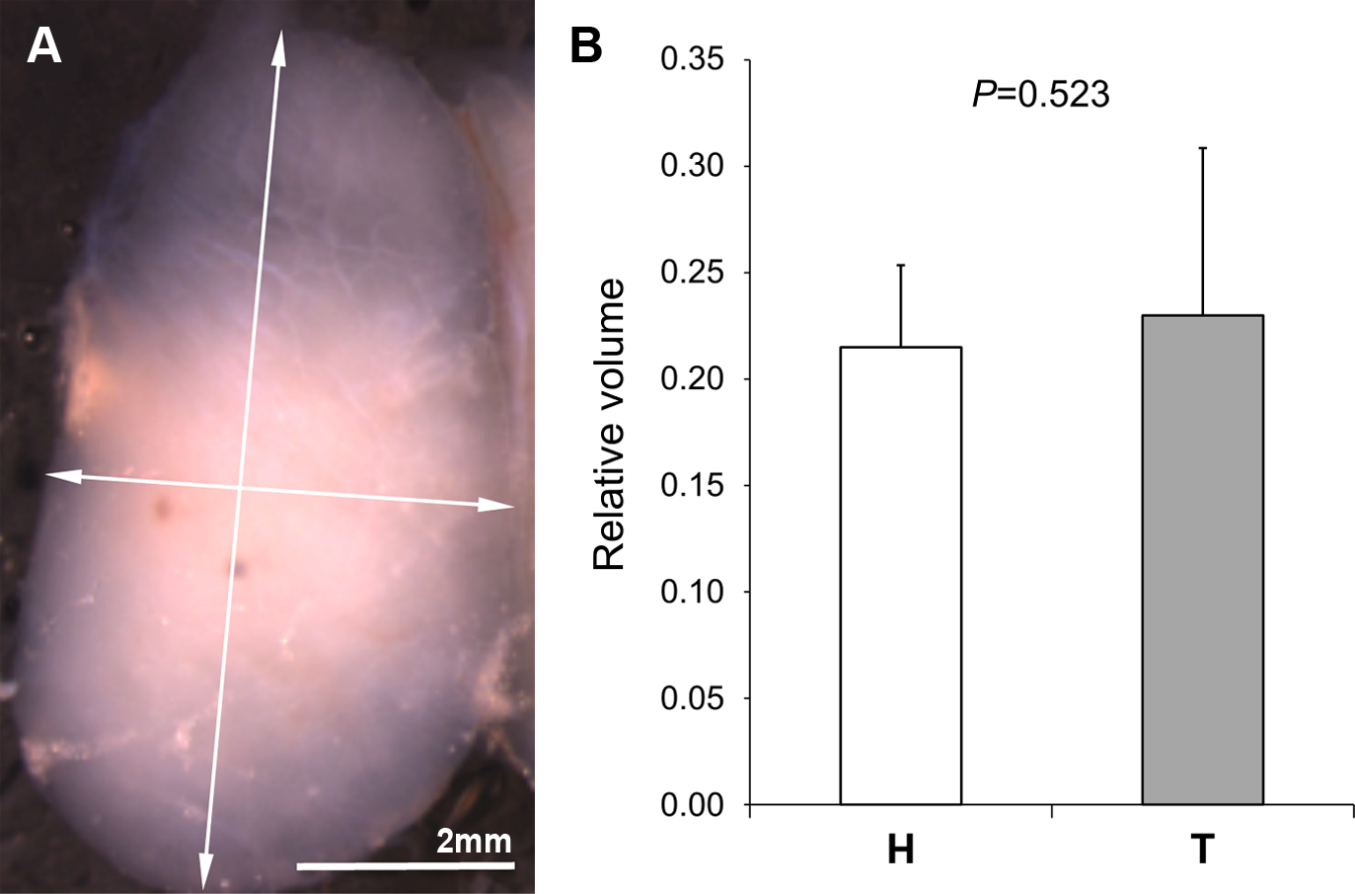
Anatomy and size of the thymus. (A) Frontal view of the right thymic lobe of a specimen from the T strain. The arrows indicate the major and minor diameters used to estimate the volume. (B) Mean relative volume of the thymus of specimens of the H (n = 15) and the T (n = 11) strains. No statistically significant difference was found.

The thymuses from the animals of both strains showed a similar anatomy. They were bilobular, with each lobe showing an oval shape, homogeneous white color, and consistent appearance (Fig 7A). No significant difference in volume was found between the right and left thymic lobes in animals of the H (*p* = 0.378) and T (*p* = 0.233) strains. Under the binocular microscope, the thymuses from the animals of the H strain appeared bigger than those from the T strain. However, differences in the relative mean thymic volume between animals of the H and T strains (Fig 7B) were not significant (*p* = 0.523).

### Thymus. Hassall’s corpuscles

**Fig 8:**
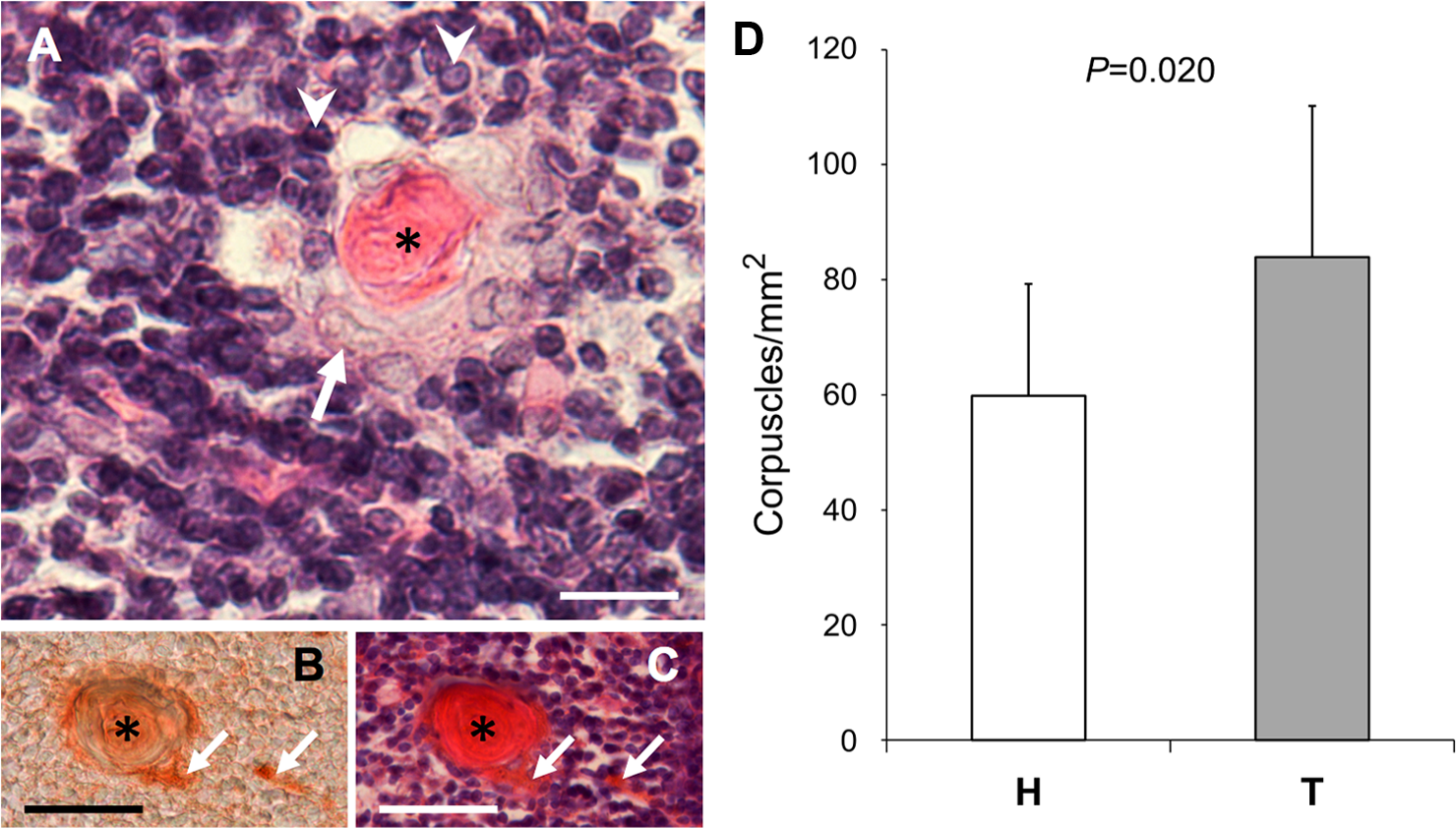
Hassall’s corpuscles. (A-C) Micrographs of the thymus stained with HE (A and C) or immunostained using KRT10 antibodies (B). B and C correspond to consecutive sections. The asterisks mark the Hassall’s corpuscles, the arrows point to epithelial reticular cells, and the arrowheads to lymphocytes. Scale bars: 17 μm (in A); 50 μm (in B and C). (D) Mean density of Hassall’s corpuscles in animals of the H (n = 15) and T (n = 11) strains. A significant (*p* = 0.020) 0.71-fold increase was found in the T strain.

The general histological structure of the thymus was similar in the animals of the two strains. HE staining allowed the identification of three basic components of the thymus, i.e. Hassall’s corpuscles, epithelial reticular cells, and lymphocytes (Fig 8A). Hassall’s corpuscles were easily distinguishable due to their eosinophilic central mass, which was highly reactive to KRT10 antibodies (Fig 8B and 8C).

No significant difference was detected in the density of Hassall’s corpuscles between the right and left thymic lobes in animals of both the H (*p* = 0.495) and T (*p* = 0.880) strains. When measurements of both lobes of each animal were computed together, differences in the density of Hassall’s corpuscles between animals of the H and the T strains were significant (*p* = 0.020); a 0.71-fold increase in the density of Hassall’s corpuscles was found in the T strain (Fig 8D).

## Discussion

Currently, the T strain of Syrian hamsters is the only spontaneous animal model of BAV disease. In this model, A-P BAV is an isolated, non-syndromic defect, i.e. it is not associated with other major cardiac or extracardiac malformations. Although hamsters with BAV do not develop the associated complications frequently found in human patients, i.e. valvulopathies and aorthopathies, the anatomy and the inheritance pattern of this valvular defect are similar in both species. Thus, the aforementioned strain is considered a well-established model of the human isolated BAV disease [8,9,66,67]. Embryological studies of the formation of A-P BAV, conducted with this model, pointed to anomalies of the NC as the etiological trigger, although the specific morphogenetical mechanisms leading to valve maldevelopment are still unknown [12].

The results of the present study uncover the existence of non-pathological changes of both the shape of the mandible and the histomorphology of the thymus in the hamster model of isolated BAV disease. Compared to controls, the hamsters of the affected strain show, in general terms, a significantly smaller mandible, significantly different mandible shape, and a significant increase in the density of Hassall’s corpuscles of the thymus. Since the specimens of the H and T strains were reared under the same laboratory conditions, the phenotypic differences detected in the present study can be attributed to differences in the genetic background derived from inbreeding. Two alternative hypotheses may explain the combined occurrence of specific valvular, mandibular, and thymic phenotypes in the T strain: 1) the phenotypes were randomly selected across inbreeding generations; 2) the phenotypes share a common developmental program. Our results point to the second hypothesis, at least with regard to the association between aortic valve and mandible phenotypes. When considering the whole sample, significant mandible shape differences were found between specimens with BAV and TAV irrespective of the strain.

In the control strain, significant mandible shape differences were found between individuals without (TAV-0), with little (TAV-1) or with large (TAV-2) fusion of the valve leaflets; major differences were detected between the latter and the first two groups. In the T strain, hamsters without fusion of the valve leaflets (TAV-0) clearly differed from the rest of the groups established in accordance with the morphological variants of TAV and BAV. Likewise, in this strain, mandible shape of the individuals with BAV differed significantly among the groups established according to the size of the raphe (BAV-3, BAV-4, and BAV-5). These results suggest that, independently of the inbreeding, there is a developmental association between aortic valve and mandible phenotypes.

Many traits, instead of showing discrete phenotypes, vary continuously over a range of phenotypic values. Such continuous variability arises because of complex patterns of action and interactions among a number of independently segregating genetic factors. These factors may be expressed differentially during ontogeny, and their expression is often modulated significantly by heritable epigenetic effects as well as by nonheritable environmental factors. This is probably the case of the aortic valve. Although the traditional clinical view considered human TAVs and BAVs as discrete phenotypes, a number of studies have shown that there exists a continuous range of valve phenotypes [11,68–70]. In the hamster, the variation of the aortic valve morphology ranges from TAVs without fusion of the leaflets to BAVs devoid of any raphe [50–53]. This continuous range of phenotypes is the result of a complex, polygenic pattern of inheritance, with variable expressivity and reduced penetrance, probably modulated by epigenetic factors and intangible variation, or developmental noise [51,53]. As for mandible shape, the main differences between strains were found in the ascending ramus, which is ontogenetically more complex than the alveolar region. In fact, the development and action of the masticatory muscles that insert onto the posterior half of the dentary highly contribute to the phenotypic variation of this region. Further, this higher ontogenetic complexity of the ascending ramus is probably related to the fact that this trait tends to be more highly heritable [71,72] and controlled by a larger set of quantitative trait loci [37,73], in comparison to the alveolar region. Different developmental factors related to cell population dynamics are involved in the assemblage of the mandible, and an alteration of any of these factors may produce developmental and evolutionary changes. According to Atchley and Hall (1991) [31], the developmental and evolutionary phenotypic variability of the mandible depends on the additive intrinsic genetic variance, the genetic variance due to epigenetic effects, the prenatal and postnatal maternal effects, and the residual nonheritable environmental effects. Thus, the complex nature of the etiological factors affecting the morphology of the aortic valve and the ascending ramus, including genetic variance, epigenetic modulators, and developmental noise, associated with the behavior of the NCCs, fits well with our proposal of a common developmental pathway involving the morphology of the aortic valve and the mandible.

While most of the skull and the maxilla derive from the most rostral CrNCCs at the level of the midbrain, the mesenchymal derivatives of the mandible, neck, and pharyngeal organs such as the thymus and the parathyroid glands derive from the CrNCCs at the hindbrain level [20,21]. The CaNCCs, which are the most caudal CrNCCs, contribute to the development of cardiovascular structures such as the cardiac outflow tract, including the aortic valve, and the arteries of the aortic arch system [19,21,22,74]. Given that the morphology of the aortic valve, the anatomical components of the mandible, and the Hassall’s corpuscles of the thymus directly depend on migration and differentiation of CrNCCs at different rostro-caudal levels, our results indicate that the occurrence of aortic valve, mandible, and thymus alterations in our hamster model is the consequence of a primary defect in the CrNCCs, prior to their migration into target embryonic tissues. This morphogenetic association corresponds with the concept of polytopic syndrome [75,76], where affected organs are linked together in some intercellular developmental pathway. One example is the *Hoxa1* null mouse [29]. Absence of *Hoxa1*, which is normally expressed in CrNCCs, but not in the NC target tissues, causes a defective specification of CrNCCs, leading to alterations of the thymus, parathyroid glands, great arteries, and cardiac outflow tract, including BAV. A typical example of a polytopic syndrome in humans is the DiGeorge syndrome [77], which includes defects of the thymus, parathyroid glands, craniofacial structures, great arteries and cardiac outflow tract, caused by alterations of the NC embryonic field. Interestingly, BAV is a common feature in DiGeorge syndrome. Thus, we propose that isolated, non-syndromic A-P BAV formation is the consequence of early alterations of CrNCCs specification or migration, and not of local defects of the CaNCCs colonizing the cardiac outflow tract.

The association of non-pathological defects of several CrNCCs derivatives such as the aortic valve, the mandible, and the thymus suggests that these alterations may constitute the *forme fruste* of a polytopic syndrome. Similar hypotheses have been previously formulated in the context of BAV association with other cardiac and extracardiac anomalies [5,78]. The higher rate of BAV in families with left ventricular outflow tract (LVOT) malformations (hypoplastic left ventricle, aortic valve stenosis, coarctation of the aorta, interrupted aortic arch) compared to the general population led to the proposal that BAV is a mild manifestation of the more serious LVOT malformations [79–81]. In addition, the high incidence of BAV in patients with coarctation of the aorta and anomalies of the head/neck structures, and their relatives, led to the hypothesis that BAV is the expression of a developmental defect that affects cardiac and extracardiac NC derivatives [5].

In summary, our results indicate that in the hamster model of BAV disease there is a developmental association among the formation of BAV, the morphology of the mandible, and the differentiation of the Hassall’s corpuscles. This suggests that the formation of isolated BAV, at least A-P BAV, is the consequence of early alterations of CrNCCs specification or migration, and not of local defects of the CaNCCs in the cardiac outflow tract. Our results support the hypothesis that A-P BAV, in the hamster model and in a proportion of affected patients, may be the *forme fruste* of a polytopic syndrome involving the CrNCCs.

## Supporting information

S1 Table Raw coordinates of the 19 landmarks digitized in the sample analyzed. (XLSX)
